# Evaluation of Patient Satisfaction of an Outpatient Colonoscopy Service in an Asian Tertiary Care Hospital

**DOI:** 10.1155/2012/561893

**Published:** 2012-04-23

**Authors:** Wah-Kheong Chan, Khean-Lee Goh

**Affiliations:** Division of Gastroenterology, Department of Medicine, Faculty of Medicine, University of Malaya, 50603 Kuala Lumpur, Malaysia

## Abstract

*Aim*. To evaluate patient satisfaction towards an outpatient colonoscopy service and analyze areas of dissatisfaction for potential improvement. *Method*. Consecutive patients attending the outpatient diagnostic colonoscopy service in University of Malaya Medical Centre between 1st February and 31th July 2010 were interviewed using a questionnaire modified from the modified Group Health Association of America-9 (mGHAA-9) questionnaire. Favorable/unfavorable responses to each question, contribution of each question to unfavorable responses, and effect of waiting times on favorable/unfavorable response rates were analyzed. *Results*. Interview was carried out on 426 patients (52.1% men). Mean age ± standard deviation was 61.3 ± 12.9 years old. Mean waiting times for colonoscopy appointment and on colonoscopy day were 3.8 ± 2.7 months and 1.1 ± 0.8 hours, respectively. The main factors that contributed to unfavorable responses were bowel preparation followed by waiting times for colonoscopy appointment and on colonoscopy day (32.3%, 27.5%, and 19.6%, resp.). Favorable responses diminished to undesirable levels when waiting times for colonoscopy appointment and on colonoscopy day exceeded 1 month and 1 hour, respectively. *Conclusion*. Bowel preparation and waiting times were main factors for patient dissatisfaction. Waiting times for colonoscopy appointment and on colonoscopy day should not exceed 1 month and 1 hour, respectively, to maintain acceptable levels of patient satisfaction.

## 1. Introduction

The incidence of colorectal cancer is rapidly increasing in the Asia-Pacific region [1]. Colonoscopy remains the most accurate tool in diagnosing this condition and is now advocated in many regions to be the modality of choice for screening and surveillance [2]. Apart from visual diagnostic capabilities, it allows tissue sampling for histological confirmation and offers therapy in the form of polyp and early cancer resection [3].

However, there are various barriers toward patient acceptance of colonoscopy whether in the context of colorectal cancer screening and surveillance or even as an investigational tool in symptomatic patients. One such barrier pertains to patient dissatisfaction towards the procedure. Patients who are dissatisfied are less likely to comply with management plan or more reluctant to continue utilizing a particular healthcare service [4].

For example, discomfort during bowel preparation and discomfort during colonoscopy, factors which may be related to dissatisfaction towards the procedure, have been recognized as two of the most important deterrents for screening colonoscopy regardless of among screened or never-screened patients [5]. Another example is waiting times for colonoscopy appointment and on the colonoscopy day which have also been recognized as major factors for patient dissatisfaction towards their experience with the procedure [6, 7].

Factors contributing to patient dissatisfaction may vary with different populations. For example, in a Canadian study, Ko et al. [8] did not find waiting times for colonoscopy appointment and on colonoscopy day to affect patient satisfaction although this was the case in other centers [6, 7]. As there are limited published studies on patient satisfaction towards colonoscopy from Asian populations, we aimed to identify and report main factors for patient dissatisfaction towards the outpatient colonoscopy service in an Asian tertiary care hospital and to analyze areas of dissatisfaction for potential improvement.

### 1.1. Outpatient Colonoscopy Service in University of Malaya Medical Center

University of Malaya Medical Centre is a 1200-bedded university hospital which functions as a general-type hospital serving a mainly residential suburban area of Kuala Lumpur which is the capital city of Malaysia. Our center provides inpatient and outpatient diagnostic (including screening and surveillance) and therapeutic colonoscopy services. Our center practices an open-access outpatient colonoscopy service receiving patients from primary care clinics, other specialist clinics, and those discharged from inpatient wards in addition to patients from the gastroenterology clinic. Colonoscopy appointments are given on a first-come-first-serve basis. When a patient is deemed to require an earlier colonoscopy appointment, the doctor in charge would negotiate the patient's appointment to an earlier date on a case-to-case basis.

Patients receive instructions on bowel preparation on the day the appointment is given. A standardized bowel preparation regime consisting of polyethylene glycol electrolyte lavage solution (PEG-ELS) (Fortrans) and bisacodyl is used for all patients. Bowel preparation starts three days prior to scheduled colonoscopy appointment. Patients will take two tablets of bisacodyl at 2000H on D1. Patient should be on low-residue diet from D2 onwards and will take another 2 tablets of bisacodyl at 2000H on D2. Patients will take 2 liters of Fortrans within an hour from 1800H till 1900H on D3. Patients are allowed plain water only till colonoscopy once they start taking Fortrans. For patients whose colonoscopy is scheduled in the afternoon, Fortrans is taken within an hour from 0800H till 0900H on colonoscopy day.

Appointment time on colonoscopy day is staggered half hour per patient to reduce waiting time. A support staff will register patients and a staff nurse will help patients change their dress for the procedure. All patients receive a combination of Midazolam 2.5 mg to 5 mg and Pethidine 25 mg to 50 mg or Fentanyl 50 *μ*g to 100 *μ*g as sedation prior to the procedure. Colonoscopy is performed by various grades of endoscopist including consultants, specialists, and trainees under supervision. Following the procedure, patients rest in the recovery area until they regain full consciousness before they are seen by the endoscopist in charge at the discharge counter who would explain the colonoscopy findings to them before they go home.

## 2. Methods

This is a single-center patient satisfaction survey using an on-site investigator-administered questionnaire of consecutive patients attending the outpatient diagnostic (including screening and surveillance) colonoscopy service in University of Malaya Medical Center between 1st February and 31thJuly 2010.The questionnaire used was based on the modified Group Health Association of America-9 (mGHAA-9) questionnaire but with the question on technical skill of endoscopist being replaced by a question on patient comfort level during endoscopy and the addition of a question on comfort level during bowel preparation. The items in the questionnaire used are as follows: Q1—length of time spent waiting for the appointment—Q2—length of time spent waiting at the Endoscopy Suite for the procedure—Q3—comfort level during bowel preparation—Q4—personal manner of the physician who performed the procedure—Q5—personal manner of the nurses and other support staff—Q6—adequacy of explanation of what was done for you—Q7—comfort level during the procedure—Q8—overall rating of the visit—Q9—would you have the procedure done again by this physician? Q10—would you have the procedure done again at this facility? The original ordinal five-value Likert scale (excellent, very good, good, fair, and poor) was used.

Additional information on patient characteristics (age, gender, ethnicity), whether colonoscopy was surveillance or nonsurveillance, duration of waiting time for colonoscopy appointment, and duration of waiting time on colonoscopy day were obtained. Colonoscopy was categorized as surveillance if a patient already had a colonoscopy previously and the repeat colonoscopy was for surveillance where a predetermined interval from the last colonoscopy was intended. These patients would be aware of the intended interval from their last colonoscopy and would unlikely give unfavorable response to waiting time for colonoscopy appointment and were therefore excluded in the analysis of data on waiting time for colonoscopy appointment. On the other hand, colonoscopy was categorized as non-surveillance if it was the patient's first colonoscopy, whether it was a screening colonoscopy in an asymptomatic patient or a diagnostic colonoscopy in a symptomatic patient. Waiting time for colonoscopy appointment refers to the duration from the day the procedure was planned to the day that it was performed while waiting time on colonoscopy day refers to the duration from the time of registration on the day of the procedure to the time the procedure was performed. The first twenty patients interviewed were also asked an open-ended question regarding any aspects that they were dissatisfied with that were not covered in the questionnaire.

Face-to-face interview based on the questionnaire was conducted after patients have recovered from sedation and given explanation about their colonoscopy findings just before they left the Endoscopy Suite. The investigators who interviewed the patients were not involved in any aspects of the care of the patient on colonoscopy day. The investigators introduced themselves as research personnel who are conducting a patient satisfaction survey. Patients were encouraged to give an honest response to each of the questions in the questionnaire with the reassurance that their identity and responses will remain confidential. Informed consent was obtained from all subjects before entering the study. The study conforms to the provisions of the Declaration of Helsinki 1995 and was approved by the hospital Ethics Committee.

### 2.1. Statistical Analysis

Data were analyzed using a statistical software program, Statistical Packages for the Social Sciences (SPSS) version 11.5. (Chicago, Illinois, USA). Continuous variables were expressed as mean ± SD. Waiting time for colonoscopy appointment was categorized as within 1 week, between 1 week to 1 month, between 1 month to 3 months, between 3 months to 6 months, and over 6 months. Waiting time on colonoscopy day was categorized as within half hour, between half hour to 1 hour, between 1 hour to 2 hours, and over 2 hours. Patient response for each of the questions 1 to 8 was dichotomized to favorable (excellent, very good, good) and unfavorable (fair, poor). The percentages of favorable and unfavorable responses for each of the questions were calculated. A problem rate was also estimated by dividing the sum of unfavorable responses with the sum of questions asked and multiplying by 100. A Pareto chart was used to illustrate the contribution of each of the questions to the overall unfavorable responses. Finally, the percentages of favorable and unfavorable responses were estimated across the categories of waiting time for colonoscopy appointment and waiting time on colonoscopy day and analyzed using Chi-square test.

## 3. Results

### 3.1. Patient Characteristics

A total of 426 patients were interviewed consisting of 222 (52.1%) men and 204 (47.9%) women. Mean age ± standard deviation of the study population was 61.3 ± 12.9 years old. Majority of patients were Chinese (63.8%) followed by Indian (18.8%), Malay (15.3%), and other races (2.1%).

### 3.2. Waiting Times for Colonoscopy Appointment and on the Day of Colonoscopy

Ninety-five patients (22.3%) came for scheduled surveillance colonoscopies and were excluded from the analysis for waiting time for colonoscopy appointment. The mean waiting time for colonoscopy appointment in the group of 331 patients who came for non-surveillance colonoscopies was 3.8 ± 2.7 months. Around one-third of patients had their colonoscopies within 1 month from booking (10% within 1 week, 22% within 1 week–1 month, 17% within 1–3 months, 33% within 3–6 months, and 18% over 6 months). Mean waiting time on colonoscopy day was 1.1 ± 0.8 hours. More than two-thirds of patients had their colonoscopies within 1 hour from registration (35% within 1/2 hour, 34% within 1/2–1 hour, 27% within 1-2 hours, and 4% over 2 hours).

### 3.3. Patient Response

**Figure 1:**
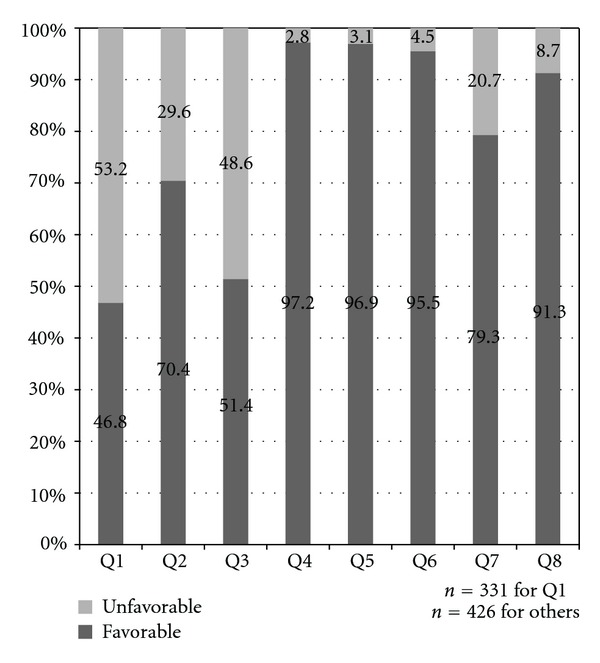
Patient responses for Q1 to Q8.

The percentages of favorable and unfavorable responses for each of the questions are shown in Figure 1. A high rate of unfavorable response was seen for waiting time for colonoscopy appointment (53.2%) and comfort level during bowel preparation (48.6%). The rate of unfavorable response for waiting time on colonoscopy day and for comfort level during colonoscopy was 29.6% and 20.7%, respectively. Most patients gave favorable response to personal manner of physician (97.2%), personal manner of nurses and support staff (96.9%), and explanation about colonoscopy findings (95.5%). The overall rating was favorable in the majority of patients (91.3%). Almost all patients would choose to return to the same hospital (99.8%) and to the same physician (97.7%) if they require a repeat colonoscopy in the future. On open-ended questioning of the first twenty patients, no additional factors for dissatisfaction were identified.

### 3.4. Main Causes of Unfavorable Responses

**Figure 2:**
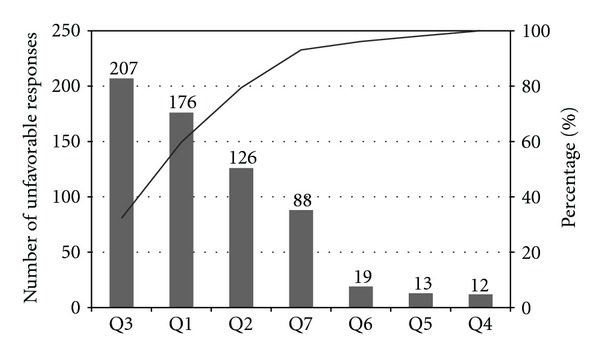
Pareto chart showing the contribution of each of the questions to unfavorable responses. The bars represent the number of unfavorable responses for each of the questions Q1 to Q7 (total number of unfavorable responses = 641). The black line represents the accumulated percentage.

The problem rate was 22.2% (641 unfavorable responses out of 2887 questions asked). The main factors that contributed to unfavorable responses were comfort level during bowel preparation followed by waiting time for colonoscopy appointment and waiting time on colonoscopy day (Figure 2).

### 3.5. Favorable Response Diminished to Undesirable Levels When Waiting Time for Colonoscopy Appointment Exceeded 1 Month

**Figure 3:**
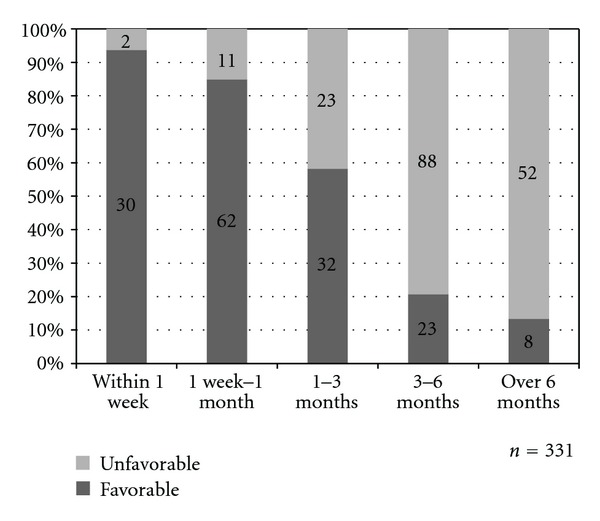
Patient response towards waiting time for colonoscopy appointment across the different duration of waiting time for colonoscopy appointment (*P* < 0.001 across each of the categories of waiting time).

Favorable response significantly decreased as waiting time for colonoscopy appointment became longer across categories of waiting time (Figure 3) (*P* < 0.001 across each of the categories of waiting time). A good rate of favorable response (87.6%) was seen when waiting time for colonoscopy appointment was within 1 month but this rate fell to 58.2% when appointment was between 1 to 3 months.

### 3.6. Favorable Response Diminished to Undesirable Levels When Waiting Time on Colonoscopy Day Exceeded 1 Hour

**Figure 4:**
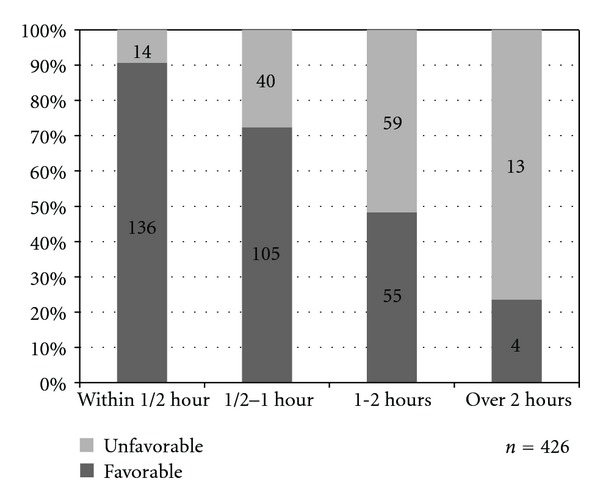
Patient response towards waiting time on colonoscopy day across the different duration of waiting time on colonoscopy day (*P* < 0.001 across each of the categories of waiting time).

Favorable response also significantly decreased as waiting time on colonoscopy day became longer across categories of waiting time (Figure 4) (*P* < 0.001 across each of the categories of waiting time). A modest rate of favorable response (81.7%) was seen when waiting time on colonoscopy day was within 1 hour but this rate fell to 55% when the waiting time was between 1 to 2 hours.

## 4. Discussion

 Evaluation of patient satisfaction and addressing areas of dissatisfaction is an important aspect of healthcare services and is a measure of quality of service provided. This process has been found to be useful in improving standards of endoscopy centers including performance of endoscopists, and possibly the reputation of endoscopy centers in the long run [9]. Lin had echoed the importance of measuring and improving patient satisfaction, describing this aspect as of prime importance for the economic future of gastrointestinal endoscopy and for gastrointestinal endoscopy to remain competitive against rival technologies [10]. Patient satisfaction also affects perception of the population at large towards endoscopic services as a whole and can have significant impact on patient willingness to undergo endoscopic procedures regardless of whether the patient has had endoscopy before.

 Different questionnaires have been used to assess patient satisfaction towards gastrointestinal endoscopy [11–13]. The American Society of Gastrointestinal Endoscopists (ASGE) recommended the use of the mGHAA-9 questionnaire to measure patient satisfaction [11]. However, mGHAA-9 does not contain a question on patient comfort which has been found to be an important factor influencing patient satisfaction [14]. It was also noted that patients had difficulty answering the question on technical skills of endoscopist found in mGHAA-9 [12]. We anticipated a similar problem with our patients and have substituted this question with one on patient comfort. In addition, we included a question on bowel preparation as we felt that this is an important aspect of colonoscopy that patients may be dissatisfied with. It was reported in a study by Yacavone et al. that a significant percentage of patients found bowel preparation negatively impacting their satisfaction towards colonoscopy [14]. Furthermore, the response obtained by Yacavone et al. was through open-ended questioning and not part of their 15-item questionnaire meaning that the true significance of negative impact of bowel preparation towards patient satisfaction could have been underestimated. To our best knowledge, this is the first time a question on bowel preparation is included in the mGHAA-9 questionnaire. Interestingly, we found discomfort during bowel preparation to be the leading cause of unfavorable responses among patients attending our outpatient colonoscopy service and propose that this item be included in future studies evaluating patient satisfaction towards colonoscopy.

Although the bowel preparation regime that we used (reduced volume PEG-ELS) has been shown to be better tolerated compared to 4-liter PEG-ELS [15], nearly half of our patients gave unfavorable response towards their bowel preparation experience. Discomfort promotes nonadherence to bowel preparation and leads to inadequate colon cleansing which could in turn adversely affect diagnostic yield and technical performance [16]. A separate study in our center [17] showed that 23.6% of patients failed to comply with bowel preparation instructions and poor quality bowel preparation was seen in 30.1%. Colonoscopic examinations of these patients were associated with increased technical difficulty and patient discomfort. Discomfort during bowel preparation has also been shown to be a major deterrent for patients to undergo colonoscopy regardless of whether they have or have never undergone colonoscopy before [5]. Improving patient comfort during bowel preparation is therefore imperative not only to ensure compliance but also to enhance patient receptiveness towards colonoscopy. Split-dose PEG-ELS has been reported to be better tolerated with significantly lower discontinuation due to adverse effects compared to conventional single-dose PEG-ELS [18] and may be helpful in addressing the issue of patient discomfort with bowel preparation faced by our centre. Moreover, split-dose PEG-ELS has been shown to provide superior colon cleansing [18, 19].

Large number of patients scheduled for colonoscopy and limited resources have resulted in long appointment waiting times in our center while prolonged waiting on the day of colonoscopy may be the result of combination of factors including overscheduling of cases for each session. More than half of our patients were dissatisfied with waiting time for colonoscopy appointment while close to one-third were unhappy with their waiting on colonoscopy day. As dissatisfaction towards appointment waiting time could have resulted in a proportion of patients transferring to another outpatient colonoscopy service, our figure could be an underestimation of the true proportion of patients who were dissatisfied in this aspect. Waiting times for endoscopy appointment and on endoscopy day are problems not restricted to our center but appear to be major causes of unfavorable responses in other centers as well [6, 7, 20, 21]. Prolonged colonoscopy appointment waiting time may reduce patient motivation to keep to their appointment and to adhere to bowel preparation instructions. In fact, prolonged colonoscopy appointment waiting time has been recognized as an independent risk factor for poor quality bowel preparation in our center [17]. In this aspect, it is vital that increasing patient load is matched by increasing allocation of resources to maintain a service that meets the expectations of not only patients but also of healthcare providers.

Besides bowel preparation experience and waiting times, other factors have yielded unfavorable responses from our patients. However, utilizing the principle of “vital few and trivial many” [22], we identified that discomfort during bowel preparation and waiting times constituted to nearly 80% of the problems faced by our patients. By focusing on improvement in these aspects, there is great likelihood of substantially reducing the problem rate among patients attending our outpatient colonoscopy service. Based on our analysis, aiming for colonoscopy appointment waiting time of within 1 month and waiting time on colonoscopy day of within 1 hour will result in an improved rate of favorable response of over 80% in these two aspects. However, as this is a single-center study, this result may not be generalizable to other populations. Nevertheless, by using a similar approach, other centers may be able to gauge the waiting times that are acceptable for their patient population.

Despite our efforts, this study has several limitations. First, the modified questionnaire that we used has not been formally validated, except for obvious face validity. Secondly, it is possible that other factors which may adversely impact patient satisfaction were unaccounted for in our study. For example, we did not include physical environment as an item in the questionnaire although this has been found to be associated with patient satisfaction [8]. However, we were satisfied that the modified mGHAA-9 questionnaire that we used in this study has covered the most important factors since no additional factors were brought up by patients when additionally asked in an open-ended manner on other aspects of dissatisfaction at the beginning of the study. Third, we concentrated on procedure-related factors and did not look into patient-related factors in our study as we felt that the former were at least partially under our control and therefore could provide more opportunities for improvements than the latter.

Although this is a single-center study, it complements well with the existing literature as there are currently limited published studies on this matter from this part of the world. Our center practices an open-access outpatient colonoscopy service and approximately 40% of patients scheduled for colonoscopy are from the primary care clinics attached to this institution [23]. Hence data from this study is generalizable to local populations scheduled for colonoscopy in general. Consecutive, instead of random, sampling was used to maximize the number of subjects recruited within the study period. It is acceptable to use consecutive sampling, which happens to be the best choice of nonprobability sampling. A good representation of the overall population was possible by studying all subjects.

It has been found that different methods of evaluation of patient satisfaction at different times may yield significantly different responses. For example, responses tend to be better when interviews were conducted on-site immediately after endoscopy or even on phone-back after a short period of time following endoscopy as opposed to when they were conducted through mail-back after a prolonged interval [8, 13]. Interesting terms such as “social desirability response” bias and “ingratiating response” bias have been used for this phenomenon where satisfaction scores were better when obtained through more personal and earlier communications with patients [10]. This factor should be considered when comparing the results of satisfaction survey between centers or between two time points in the same center but may not be a strong reason to reject on-site interviews given the higher response rate of such method and its ease of administration.

In conclusion, we found bowel preparation to be the leading cause of patient dissatisfaction of the outpatient colonoscopy service in an Asian tertiary care hospital, followed by waiting times for colonoscopy appointment and on colonoscopy day. Waiting times for colonoscopy appointment and on colonoscopy day should not exceed 1 month and 1 hour, respectively, as favorable responses diminished to undesirable levels beyond these waiting times.
